# Participation in Physical Activity and Physical Education in School Among Children With Acute Lymphoblastic Leukemia After Intensive Chemotherapy

**DOI:** 10.3389/fped.2019.00073

**Published:** 2019-03-18

**Authors:** Hamidah Alias, Nur Adlina Mohd Nazi, Doris Lau Sie Chong

**Affiliations:** Department of Pediatrics, UKM Medical Center, Faculty of Medicine, The National University of Malaysia, Kuala Lumpur, Malaysia

**Keywords:** acute lymphoblastic leukemia, physical activity, physical education in school, off-chemotherapy, barriers

## Abstract

**Background:** Low physical activity (PA) level has been reported among survivors of childhood acute lymphoblastic leukemia (ALL). The present study was performed to determine the level of participation in general PA and physical education in school (PES) among children with ALL who completed intensive chemotherapy and identify possible barriers that influence adherence to PA and PES.

**Methods:** A cross-sectional, single-center study was conducted over 1 year in a tertiary pediatric hematology and oncology referral center in Kuala Lumpur, Malaysia. A total of 47 children with ALL aged 7–18 years old who were off-treatment and attended school on a regular basis were recruited. A modified structured questionnaire adapted from the Youth Risk Behavior Surveillance System, Division of Adolescent and School Health, the Centers for Disease Control and Prevention (CDC) was used to assess the children's level of PA and PES participation.

**Results:** Among the 47 children will ALL included herein, 11 (23.4%) were physically active for at least 60 min a day for 5 days or more, following CDC recommendations. The median duration from completion of intensive chemotherapy was 4.95 years (25th, 3.29; 75th, 7.95). Younger age at study entry (median, 8.7 years old vs. 12.2 years old) and younger age at diagnosis (median, 2.9 years old vs. 4.3 years old) were significantly associated with higher PA level. Almost all children (45/47, 95.7%) participated in PES. Barriers to non-participation in PES mainly included exhaustion or fear of injury.

**Conclusions:** Majority of the children with ALL included herein had low levels of daily PA after intensive chemotherapy. Nonetheless, their participation in PES was encouraging. PA should thus be promoted during and after cessation of ALL treatment to prevent long-term health risks and improve overall quality of life.

## Introduction

Acute lymphoblastic leukemia (ALL), the most common childhood malignancy, has a 5 year overall survival of over 80% in developed countries (1, 2). Despite improved outcome, ALL survivors may experience long-term adverse effects of which some could be debilitating. Previous studies had reported that 62.3% of childhood cancer survivors suffered from at least one chronic condition, such as type 2 diabetes mellitus, obesity, and cardiovascular disease (2–6), one of the risk factors for such conditions being physical inactivity (3–7). Children with ALL have been reported to experience muscle strength deficits during and after treatment that could lead to deficits in physical activity (PA) (8). Dexamethasone-induced myopathy and vincristine-related peripheral neuropathy have also been shown to contribute toward the reduction in PA during and after completion of chemotherapy (9, 10). Previous studies had reported that childhood ALL survivors have a tendency to develop metabolic syndrome as a consequence of becoming overweight or obese due to reduced PA among other reasons (11–15).

Regular PA participation has been shown to induce protective effects by increasing muscle mass and improving strength and endurance, immune response, circulating hormones, and energy balance (16). According to a childhood cancer survivor cohort study, those with increased PA level have lower subcutaneous and body fat mass and greater lean body mass than those with low levels of PA (17). Moreover, the Centers for Disease Control and Prevention (CDC) has recommended that individuals over 18 years old should engage in 30 min of moderate to vigorous activity for at least 5 days a week (18). For children and adolescents below 18 years old, 60 min of moderate to vigorous activity at least 5 days per week is recommended (18). Moderate intensity activity refers to activities similar to a brisk walk, water aerobics, and biking on level ground with a few hills, whereas vigorous intensity activity refers to activities similar to a jog or run, hiking uphill, fast dancing, rope jumping, and swimming (19). However, a large number of childhood cancer survivors did not adhere to such recommendations (19–21). Reported barriers to PA participation included fatigue, concern regarding increased risk of infection, pain, low self-esteem, lack of time, and falling behind academically (17). Moreover, physicians provided insufficient exercise counseling to their patients and families (17).

PA programs in school have been shown to increase PA level and overall fitness in children, including childhood cancer survivors (17). However, during intensive chemotherapy, most children and adolescents do not attend school or participate in any PA. Prolonged hospital stay and frequent admissions could further reduce their level of involvement in PA (22, 23). Upon returning to school, many of them may be excluded from sports activities and physical education in school (PES). Kesting et al. reported that one in 4 children previously treated for childhood cancer was not being integrated into PES (22). Common reasons reported for non-participation in PES were prohibition from parents, teachers or attending physician, presence of an *in-situ* catheter, or problems related to prosthetic devices. Considering that PES is one of the compulsory subjects in both primary and secondary schools throughout Malaysia, it could be used as an initial step to promote active PA participation among the childhood cancer survivors upon returning to school. Therefore, we aimed to determine the level of PA and PES participation among children with ALL upon completion of intensive chemotherapy and identify possible barriers that may influence PA and PES adherence.

## Materials and Methods

This cross-sectional, single-center study was carried out at a tertiary pediatric hematology and oncology center in Kuala Lumpur, Malaysia over a period of 1 year. All children aged 7–18 years diagnosed with ALL who were post-intensive chemotherapy (maintenance or off-treatment) and attended school on regular basis were eligible for the study. The exclusion criteria included refusal of consent from parents or guardians. Ethical approval was obtained from the institutional Research and Ethics Committee prior to the study. Written informed consent was obtained from the parents/guardian prior to recruitment. The children and their guardian/parents (either mother or father) were given a questionnaire to answer under the guidance of the investigators.

## Assessment Tool

A structured questionnaire was used to assess the children's level of PA and PES participation. The questionnaire was adapted from the Youth Risk Behavior Surveillance System (1991–2015), Division of Adolescent and School Health, CDC and modified to suit the local population (24). The questionnaire consisted of 30 questions divided into six sections: (i) PA in general, (ii) PA in daily life, (iii) PES, (iv) PA during leisure time outside of school, (v) availability of sports facilities, and (vi) parental perception on their child's in PA and PES participation. Parts (i) to (v) were answered by the patients under the investigator's guidance, while part (vi) was answered by the parents or guardians. The original questionnaire was translated from English to Bahasa Malaysia by a bilingual translator and back-translated into English by another translator who had not seen the original questionnaire. Both independent translators were fluent in both Bahasa Malaysia and English. The translations retained the original meaning of the questionnaire and had been checked by two resident pediatricians. The content of the Bahasa Malaysia questionnaire was validated by administering it to a selected group of patients (10 subjects). The questionnaire was determined to have a Cronbach's alpha value of 0.8, showing high internal consistency and reliability. The approximate time to complete the questionnaire was 15–20 min. In this study, a patient who engaged in 60 min of moderate to vigorous activity at least 5 days per week was considered physically active as per CDC recommendations.

## Results

### Subjects

**Figure 1:**
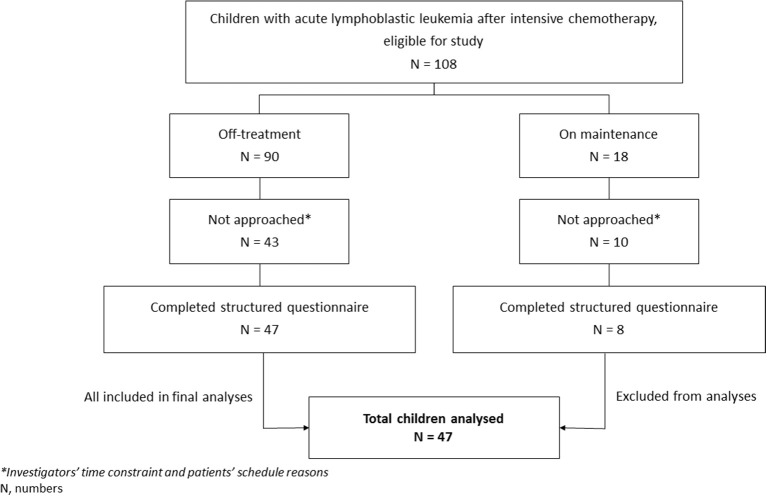
Flow chart on the recruitment of study subjects.

A total of 108 eligible children were identified from the database during the study period. Among them, 55 (50.9%) (26 males and 29 females) met the inclusion criteria, and their parents/guardian completed the questionnaire. Among the 55 children, 8 (14.5%) were on maintenance chemotherapy, while the rest were off-treatment. The remaining 53 children (49.1%) could not be recruited due to scheduling reasons and investigators' time constraints. Children on maintenance chemotherapy were later excluded from analyses given that they were too few in number. Figure 1 shows the flowchart of the study, while Table 1 shows the characteristics of the 47 off-treatment children who completed the study.

**Table 1:** Demographic and disease characteristics of the study population.

**Characteristic**	**Patients (*n* = 47)**
Age at study entry in years, median (IQR)	11.02 (25th 8.42; 75th 14.11)
**Gender**, ***n*** **(%)**	
Male	21 (44.7)
Female	26 (55.3)
**Ethnicity**, ***n*** **(%)**	
Malay	34 (72.3)
Chinese	9 (19.2)
Indian	3 (6.4)
Others	1 (2.1)
**Education level**, ***n*** **(%)**	
Primary	29 (61.7)
Secondary	18 (38.3)
Age at diagnosis in years, median (IQR)	3.53 (25th 3.06; 75th 6.15)
Duration from completion of intensive chemotherapy in years, median (IQR)	4.95 (25th 3.29; 75th 7.95)
Duration off-treatment in years, median (IQR)	3.03 (25th 1.61, 75th 6.09)
**Risk stratification**, ***n*** **(%)**	
Standard risk	36 (76.6)
High risk	11 (23.4)
**Relapse**, ***n*** **(%)**	
Yes	2 (4.3)
No	45 (95.7)
**Craniospinal irradiation**, ***n*** **(%)**	
Yes	1 (2.1)
No	46 (97.9)
**Complication during treatment**, ***n*** **(%)**	
Yes	3 (6.4)^*^
No	44 (93.6)
**Comorbidities**, ***n*** **(%)**	
Yes	5 (10.6)^*^
No	42 (89.4)
**Body mass index (kg/m**^**2**^**)**, ***n*** **(%)**	
<10th percentile	8 (17.0)
10–24.9th percentile	3 (6.4)
25–49.9th percentile	11 (23.4)
50–74.9th percentile	12 (25.5)
75–90th percentile	7 (14.9)
>90th percentile	6 (12.8)
**Fathers' education level**, ***n*** **(%)**	
Primary	1 (2.1)
Secondary	28 (59.6)
Tertiary	18 (38.3)
**Mothers' education level**, ***n*** **(%)**	
Primary	3 (6.4)
Secondary	21 (44.7)
Tertiary	23 (48.9)
**Monthly income**, ***n*** **(%)**	
<MYR1999	6 (12.8)
MYR2000–3999	16 (34.0)
>MYR4000	25 (53.2)

*n, number; IQR, interquartile range; MYR, Ringgit Malaysia*.

* *One child with underlying comorbidity also had complication during treatment*.

Among the included children, 5 (10.6%) had underlying comorbidities: two have bronchial asthma, 1 has allergic rhinitis, 1 has HbE trait, and 1 has autism spectrum disorder. Moreover, three of the 47 children (6.4%) had complications during the intensive chemotherapy: one had vincristine-induced peripheral neuropathy, 1 had bilateral femoral head avascular necrosis, and 1 had right calf abscess with concomitant right tibia fracture.

### Physical Activity

**Figure 2:**
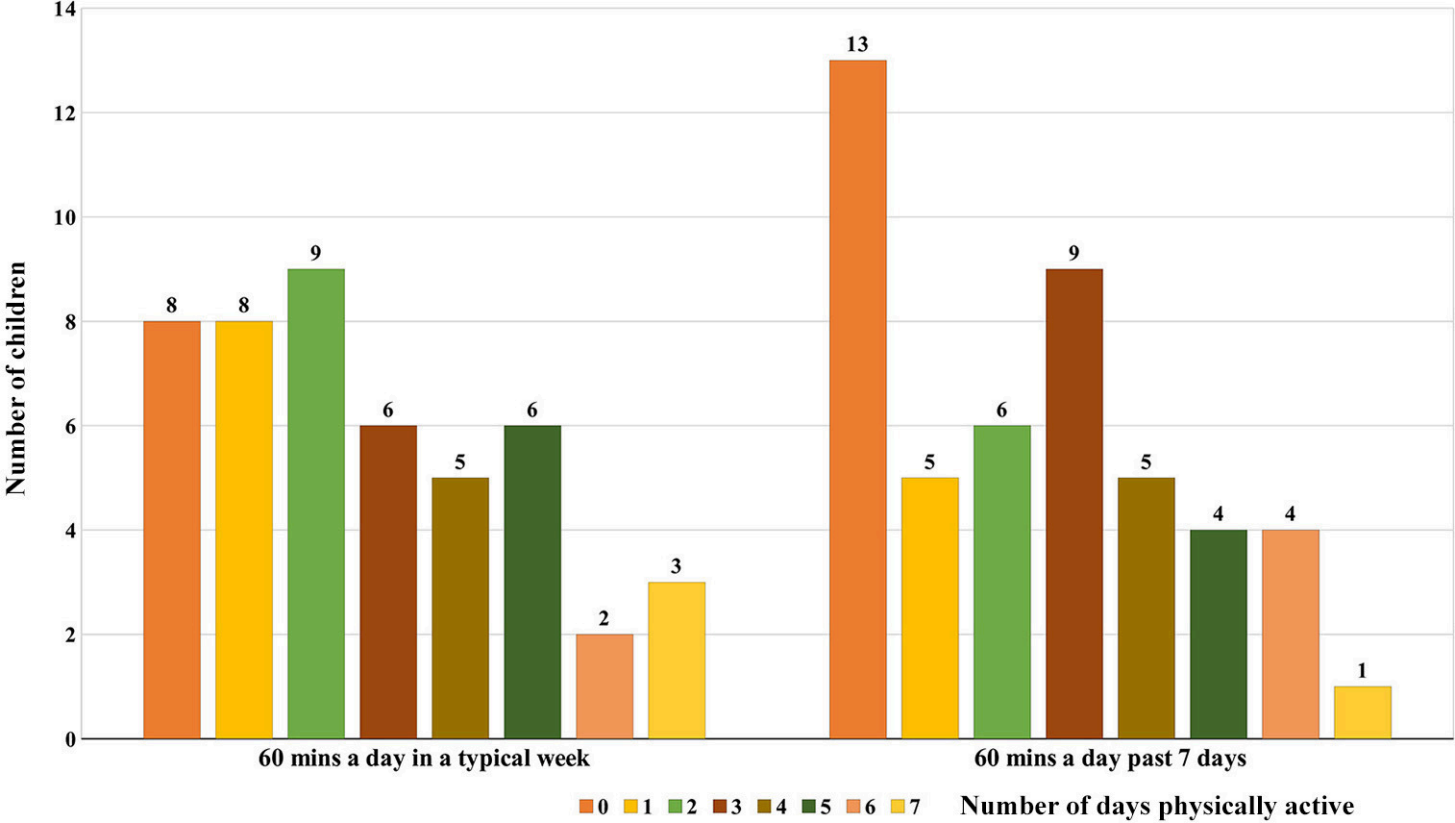
Participation of the study population in general physical activity.

In a typical week, only 11 (23.4%; 6 males and 5 females) out of the 47 children were considered physically active following CDC recommendations (Figure 2). Moreover, 20 children (42.5%) were active for at least 60 min a day for 2 days or more but <5 days a week. Assessment of PA for the past 7 days showed that only 9 (19.1%) out of the 47 children were physically active. Eight children were found to be active both during a typical week and for the past 7 days. Concordance/agreement among the study participants was strong (kappa 0.84) for the level of PA participation during a typical week and for the past 7 days. Interestingly, the child who had both asthma and vincristine-induced neuropathy during intensive chemotherapy was physically active during a typical week despite his comorbidity.

Bivariate analyses revealed that age at study entry and age at diagnosis were statistically significantly associated with PA level. Accordingly, children of younger age at study entry (median, 8.7 years old vs. 12.2 years old) and diagnosis (median, 2.9 years old vs. 4.3 years old) were noted to be more active (Table 2). Duration from completion of intensive chemotherapy, off-treatment duration, risk stratification, ethnicity, gender, body mass index (BMI), and parental education level had no significant association with PA level (Tables 2, 3).

**Table 2:** Factors affecting physical activity level among the ALL children.

	**Typical week**			**Past 7 days**		
	***N***	**Median (IQR)**	***p value***	***N***	**Median (IQR)**	***p value***
**BMI (kg/m**^**2**^**)**						
Inactive	36	18.68 (15.78; 21.77)	0.119	38	19.14 (15.82; 21.84)	0.119
Active	11	16.69 (14.00; 20.06)		9	17.00 (14.84; 19.69)	
**Age at study entry (years)**						
Inactive	36	12.16 (8.89; 14.61)	0.024^*^	38	12.19 (9.28; 15.00)	0.036^*^
Active	11	8.72 (7.33; 11.53)		9	8.79 (7.69; 11.58)	
**Age at diagnosis (years)**						
Inactive	36	4.26 (3.30; 6.59)	0.001^*^	38	4.36 (3.33; 6.61)	0.001^*^
Active	11	2.95 (2.30; 3.46)		9	3.04 (2.51; 3.46)	
**Duration off treatment (years)**						
Inactive	36	3.26 (1.18; 6.26)	0.900	38	3.03 (1.25; 5.88)	0.944
Active	11	2.98 (1.76; 4.70)		9	3.01 (1.72; 6.37)	
**Duration from completion of intensive chemotherapy (years)**						
Inactive	36	5.28 (3.23; 8.37)	0.782	38	4.95 (3.25; 8.23)	0.907
Active	11	4.76 (3.34; 6.72)		9	5.15 (3.30; 8.12)	

*Statistical analysis using Mann-Whitney U-test*.

* *Significant p < 0.05*.

**Table 3:** Factors associated with physical activity level in a typical week.

		**Physical activity level**			
		**Inactive**	**Active**		
		***n* (%)**	***n* (%)**	**χ^2^**	***p value***
Gender	Male	15 (41.7)	6 (54.5)	0.16	0.685
	Female	21 (58.3)	5 (45.5)		
Ethnicity	Malay	24 (66.7)	10 (90.9)	6.13	0.105
	Chinese	9 (25.0)	0 (0)		
	Indian	2 (5.5)	1 (9.1)		
	Others	1 (2.8)	0 (0)		
Risk stratification	Standard risk	26 (72.2)	10 (90.9)	0.76	0.382
	High risk	10 (27.8)	1 (9.1)		
Complication	Yes	2 (5.6)	1 (9.1)	0.00	1.000
	No	34 (94.4)	10 (90.9)		
Comorbidities	Yes	4 (11.1)	1 (9.1)	0.00	1.000
	No	32 (88.9)	10 (90.9)		
Education year	Primary	19 (52.8)	10 (90.9)	3.70	0.055
	Secondary	17 (47.2)	1 (9.1)		
Fathers' education	Primary/secondary	22 (61.1)	7 (63.6)	0.00	1.000
	Tertiary	14 (38.9)	4 (36.4)		
Mothers' education	Primary/secondary	17 (47.2)	7 (63.6)	0.37	0.543
	Tertiary	19 (52.8)	4 (36.4)		
Family income	<RM1999	4 (11.1)	2 (18.2)	1.65	0.438
	RM2000–3999	11 (30.6)	5 (45.4)		
	>RM4000	21 (58.3)	4 (36.4)		

*Statistical analysis using chi square test. Significant p < 0.05*.

Among the 47 children included, only 24 (51.1%) had easy access to sports facilities. Nearly half of the children (28/47; 59.6%) were members of at least one sports club, while the rest (19/47; 40.4%) were not involved in any sports club.

### Physical Education in School

Majority of the primary school children (27/29; 93.1%) and all secondary school children (18 patients) participated in PES. The 2 children who did not participate in PES were treated for high risk ALL, one of whom had history of right calf abscess with concomitant right tibia fracture during intensive chemotherapy. One child did not participate in PES due to teachers' advice, while the other one did not participate due to parental restriction.

Out of the 45 children, 28 (62.2%) started to participate in PES within 6 months of returning to school. The remaining 17 children (37.8%) only took part in PES more than 6 months after returning to school (7 children participated in PES between 6 and 12 months; 10 children participated after 1 year). The delay in PES participation was mainly due to restriction by either parents or teachers. Majority of the children participated in PES for more than 20 min per session (Table 4). Most of them (60.0%) participated in every game or exercises during the PES session with varying degree of exertion.

**Table 4:** Children's level of participation in Physical Education in School (PES).

**Level of participation**	***n* = 45**
**Duration spent on each pes session**, ***n*** **(%)**	
>30 min 20–30 min	19 (42.2%) 20 (44.5%)
10–20 min	5 (11.1%)
< 10 min	1 (2.2%)
**Type of activity**, ***n*** **(%)**	
Participate in every game/exercise	27 (60.0%)
Participate in some of the activities	18 (40.0%)
**Degree of exertion**, ***n*** **(%)**	
A lot of sweating and shortness of breath	19 (42.2%)
Slightly sweating and shortness of breath	20 (44.5%)
Without sweating and without shortness of breath	6 (13.3%)

*n, number*.

### Parental Perception and Attitude Toward PES

All parents agreed that PA and PES are important for their children's health. However, when asked retrospectively regarding their children's participation in PES during maintenance chemotherapy, only 30 of the 47 parents (63.8%) allowed their children to participate. Of the 30 parents, 9 (30.0%) only allowed their children to participate in less vigorous activities during maintenance chemotherapy. Among the reasons given for not allowing their children to participate in PES while still on maintenance chemotherapy were fear of injury during the session (47.1%), child's complaint of fatigue (29.4%), and child's low self-esteem (5.9%). However, most of the parents (35/47; 74.5%) allowed their children to participate in PES upon completion of chemotherapy. Those who disagreed cited the following reasons: fear of overfatigue or infection (66.7%), presence of chemoport (8.3%), and fear of injury (8.3%). Of the 47 parents, 22 (46.8%) agreed that the best time for their children to participate in PES was within 6 months of treatment completion. Only 6 of the 47 parents (12.8%) concurred that their child should only be integrated back into PES 1 year after off-treatment.

## Discussion

The current study found that a high proportion of children treated for ALL were not physically active after intensive chemotherapy. Almost half of the children only engaged in PA <3 days a week. Our results are similar to findings from previous studies on reduced PA in children treated for ALL after completion of intensive chemotherapy or among the survivors (15, 25, 26). Another study reported that survivors of childhood ALL had PA levels similar to healthy children who met the recommended PA levels (27). The present study found that older age at study entry was associated with low PA levels. Similar findings had been reported in other studies where a decline in PA was observed with increasing age or during transition from middle school to high school (27, 28). Moreover, one study reported that regular and vigorous PA consistently reduced from 12 years old up to 21 years old (28). Although we postulate that preference for indoor activities and sedentary behaviors (e.g., watching television or playing computer games) could have contributed to this decline, other factors, such as psychological issues or lower self-esteem, may have played a role in the low level of PA among teenagers and young adults. However, such factors had not been investigated herein. Meanwhile, the increased likelihood of younger children being involved in outdoor activities like running, climbing, jumping, and cycling is consistent with their developmental milestone of being curious and eager to explore their surroundings at this age. Younger age at diagnosis, and thus at completion of ALL treatment, could also lead to better recovery of overall health, early return to school, and active re-integration into PA and PES, all of which contribute to increased PA. To date, however, no study has reported on the correlation between younger age at diagnosis and higher level of PA participation. A larger study population would be needed to confirm this finding. No association had been observed between potential risk factors, like gender, ethnicity, children's education level, and BMI, and the level of PA.

To significantly reduce or prevent long-term health risks, our study supports the importance of promoting PA and healthy lifestyle habits among children treated for ALL. Studies have shown that exercise interventions in pediatric hematological cancer survivors promotes muscle strength and cardiorespiratory fitness, particularly if the training was conducted early in the hospital (29, 30). Tailored PA and exercise programs are important in certain subgroups of children treated for ALL to avoid injury and facilitate physical function. Thorsteinsson et al. reported that performing strenuous PA while receiving cancer treatment was safe and feasible for children and adolescents (31). Therefore, PA and physical training programs should be promoted among children with cancer immediately upon diagnosis to preserve premorbid physical function. Parents and family members should also be educated on the importance and safety of PA for their children. Furthermore, increased family engagement, especially mothers as exercise partners, plays an important role in influencing PA behavior in children (17). Other studies have also reported similar positive effects of parental participation on PA level among children (32, 33).

Although only 3 of the children included herein had complications during intensive chemotherapy, these complications involved the lower limbs (vincristine-induced peripheral neuropathy, bilateral hip avascular necrosis, and right calf abscess/tibia fracture), which could prolong their period of immobility and thus limit their PA. In this particular subgroup, a comprehensive rehabilitation program involving pediatricians, physiotherapists, occupational therapists, psychologists, and family members should be initiated as early as possible to accommodate their health-specific limitations.

Our study nevertheless showed that majority of the children participated in PES. This finding is in contrast to the report by Kesting et al. where only 25% of the children previously treated for cancer were integrated into PES (22). The difference in the outcome could have been attributed to the heterogeneity of the study cohort considering that our study only involved children with ALL, whereas that by Kesting et al. included children with other types of cancers, such as bone, brain, and other solid tumors. Therefore, a higher number of patients who could not participate in PES due to physical disability, limb amputation, and endoprosthetic replacement could have been present in Kesting et al.'s study. Parental fear of their children being injured or overfatigued during treatment should be addressed accordingly to avoid unnecessary restriction to their PA participation. Other concerns regarding the risk of infection and *in-situ* catheters could be addressed during discussion to alleviate parents' and teachers' anxiety.

## Limitations

The following are the limitations of the present study: (i) cross-sectional design with no control group, (ii) small sample size, (iii) recall bias when answering the questionnaire, (iv) items assessed in the self-report questionnaire may not reflect the actual PA level, (v) no objective measures of PA level were used, and (vi) lack of assessment on parental involvement in PA. The current study also excluded children with other cancer diagnoses. The main limitation of the present study is that PA was not objectively measured with any accelerometer. In addition, obtaining the children's attendance record for PES could have enhanced the understanding on their participation in such activities. Thus, the level of PA participation may have been under- or overestimated by the children. Future multi-center studies recruiting children with different types of cancer diagnosis or studies employing a longitudinal design would be more reflective of the PA status among the general pediatric oncology population throughout Malaysia. Nevertheless, our findings highlight the need for clinicians to recognize and address the issue of low PA participation among children with ALL. Our results also underscore the need for hospitals to create concepts and initiate programs mirroring those already established in developed countries.

## Conclusion

The current study showed that majority of the children treated for ALL had low levels of PA after completion of intensive chemotherapy. However, their participation in PES was encouraging. The next logical step would be to conduct a longitudinal study including a larger cohort of children with different types of cancers; assess PA at diagnosis, during treatment, and after cessation of treatment using an accelerometer; and compare the findings with those from healthy controls.

## Data Availability

The raw data supporting the conclusions of this manuscript will be made available by the authors, without undue reservation, to any qualified researcher.

## Ethics Statement

Name of Ethics committee: Universiti Kebangsaan Malaysia Medical Center Ethics and Research; Ethics Committee/RB ref no: UKM PPI/111/8/JEP-2016-607; Research code: FF-2016-418.

## Author Contributions

DL and HA contributed to the conception and design of the study. NM and DL organized the database and performed statistical analysis. NM, DL, and HA drafted the manuscript. All authors contributed to the revision of the manuscript and have read and approved the submitted version.

### Conflict of Interest Statement

The authors declare that the research was conducted in the absence of any commercial or financial relationships that could be construed as a potential conflict of interest.

## References

[1] Pui CH. Acute lymphoblastic leukemia in children. Curr Opin Oncol. (2000) 12:3–12. 10.1097/00001622-200001000-00002 10687723

[2] Howlader N Krapcho M Garshell J Neyman N Altekruse SF Kosary CL SEER Cancer Statistics Review, 1975–2010. Bethesda, MD: National Cancer Institute (2013).

[3] Oeffinger KC Mertens AC Sklar CA Kawashima T Hudson MM Meadows AT. Chronic health conditions in adult survivors of childhood cancer. N Engl J Med. (2006) 355:1572–82. 10.1056/NEJMsa060185 17035650

[4] Veringa SJ van Dulmen-den Broeder E Kaspers GJ Veening MA. Blood pressure and body composition in long-term survivors of childhood acute lymphoblastic leukemia. Pediatr Blood Cancer. (2012) 58:278–82. 10.1002/pbc.23251 21793179

[5] Meacham LR Sklar CA Li S Liu Q Gimpel N Yasui Y. Diabetes mellitus in long-term survivors of childhood cancer: increased risk associated with radiation therapy: a report for the childhood cancer survivor study. Arch Intern Med. (2009) 169:1381–8. 10.1001/archinternmed.2009.209 19667301 PMC3529471

[6] Mulrooney DA Yeazel MW Kawashima T Mertens AC Mitby P Stovall M. Cardiac outcomes in a cohort of adult survivors of childhood and adolescent cancer: retrospective analysis of the childhood cancer survivor study cohort. BMJ. (2009) 339:b4606. 10.1136/bmj.b4606 19996459 PMC3266843

[7] Wilson CL Stratton K Leisenring WL Oeffinger KC Nathan PC Wasilewski-Masker K. Decline in physical activity level in the childhood cancer survivor study cohort. Cancer Epidemiol Biomarkers Prev. (2014) 23:1619–27. 10.1158/1055-9965.EPI-14-0213 24842624 PMC4119523

[8] Ness KK Armenian SH Kadan-Lottick N Gurney JG. Adverse effects of treatment in childhood acute lymphoblastic leukemia: general overview and implications for long-term cardiac health. Expert Rev Hematol. (2011) 4:185–97. 10.1586/ehm.11.8 21495928 PMC3125981

[9] Jansen H Postma A Stolk RP Kamps WA Acute lymphoblastic leukemia and obesity: increased energy intake or decreased physical activity? Support Care Cancer. (2009) 17:103–6. 10.1007/s00520-008-0531-0 18989711

[10] Ness KK Hudson MM Pui CH Green DM Krull KR Huang TT. Neuromuscular impairments in adult survivors of childhood acute lymphoblastic leukemia: associations with physical performance and chemotherapy doses. Cancer. (2012) 118:828–38. 10.1002/cncr.26337 21766297 PMC3197897

[11] Davies JH Evans BA Jones E Evans WD Jenney ME Gregory JW. Osteopenia, excess adiposity and hyperleptinaemia during 2 years of treatment for childhood acute lymphoblastic leukaemia without cranial irradiation. Clin Endocrinol. (2004) 60:358–65. 10.1111/j.1365-2265.2003.01986.x 15009002

[12] Oeffinger KC Mertens AC Sklar CA Yasui Y Fears T Stovall M. Obesity in adult survivors of childhood acute lymphoblastic leukemia: a report from the childhood cancer survivor study. J Clin Oncol. (2003) 21:1359–65. 10.1200/JCO.2003.06.131 12663727

[13] Trimis G Moschovi M Papassotiriou I Chrousos G Tzortzatou-Stathopoulou F. Early indicators of dysmetabolic syndrome in young survivors of acute lymphoblastic leukemia in childhood as a target for preventing disease. J Pediatr Hematol Oncol. (2007) 29:309–14. 10.1097/MPH.0b013e318059c249 17483708

[14] Warner JT Evans WD Webb DK Gregory JW. Body composition of long-term survivors of acute lymphoblastic leukaemia. Med Pediatr Oncol. (2002) 38:165–72. 10.1002/mpo.1304 11836715

[15] Reilly JJ Ventham JC Ralston JM Donaldson M Gibson B. Reduced energy expenditure in preobese children treated for acute lymphoblastic leukemia. Pediatr Res. (1998) 44:557–62. 10.1203/00006450-199810000-00015 9773846

[16] Schrack JA Gresham G Wanigatunga AA. Understanding physical activity in cancer patients and survivors: new methodology, new challenges, and new opportunities. Cold Spring Harb Mol Case Stud. (2017) 3:a001933. 10.1101/mcs.a001933 28679694 PMC5495035

[17] Yelton L Forbis S Influences and barriers on physical activity in paediatric oncology patients. Front Pediatr. (2016) 4:131 10.3389/fped.2016.00131 28066750 PMC5165656

[18] Haskell WL Lee IM Pate RR Powell KE Blair SN Franklin BA. Physical activity and public health: updated recommendation for adults from the American college of sports medicine and the American heart association. Med Sci Sports Exerc. (2007) 39:1423–34. 10.1249/mss.0b013e3180616b27 17762377

[19] Garcia DO Thomson CA. Physical activity and cancer survivorship. Nutr Clin Pract. (2014) 29:768–79. 10.1177/0884533614551969 25335787 PMC4470419

[20] Gilliam MB Schwebel DC. Physical activity in child and adolescent cancer survivors: a review. Health Psychol Rev. (2013) 7:92–110. 10.1080/17437199.2011.603641 25484907 PMC4257474

[21] San Juan AF Wolin K Lucía A. Physical activity and pediatric cancer survivorship. Recent Results Cancer Res. (2011) 186:319–47. 10.1007/978-3-642-04231-7_14 21113771

[22] Kesting SV Götte M Seidel CC Rosenbaum D Boos J. One in four questioned children faces problems regarding reintegration into physical education at school after treatment for pediatric cancer. Pediatr Blood Cancer. (2016) 63:737–9. 10.1002/pbc.25852 26681662

[23] Fuemmeler BF Pendzich MK Clark K Lovelady C Rosoff P Blatt J. Diet, physical activity and body composition changes during the first year of treatment for childhood acute leukemia and lymphoma. J Pediatr Hematol Oncol. (2013) 35:437–43. 10.1097/MPH.0b013e318279cd3e 23211695 PMC3606649

[24] Division of Adolescent and School Health National Center for HIV/AIDS Viral Hepatitis STD and TB Prevention Centers for Disease Control and Prevention Youth Risk Behavior Surveillance System (YRBSS) Questionnaire Content National Center for (1991-2015). Available online at: www.cdc.gov/yrbs (Accessed November 1, 2016).

[25] Warner JT Bell W Webb DK Gregory JW. Daily energy expenditure and physical activity in survivors of childhood malignancy. Pediatr Res. (1998) 43:607–13. 10.1203/00006450-199805000-00008 9585006

[26] Aznar S Webster AL San Juan AF Chamorro-Viña C Maté-Muñoz JL Moral S. Physical activity during treatment in children with leukemia: a pilot study. Appl Physiol Nutr Metab. (2006) 31:407–13. 10.1139/h06-014 16900230

[27] Heath JA Ramzy JM Donath SM. Physical activity in survivors of childhood acute lymphoblastic leukaemia. J Paediatr Child Health. (2010) 46:149–53. 10.1111/j.1440-1754.2009.01653.x 20105252

[28] Caspersen CJ Pereira MA Curran KM. Changes in physical activity patterns in the United States, by sex and cross-sectional age. Med Sci Sports Exerc. (2000) 32:1601–9. 10.1097/00005768-200009000-00013 10994912

[29] Wolin KY Ruiz JR Tuchman H Lucia A Exercise in adult and pediatric haematological cancer survivors: an intervention review. Leukemia. (2010) 24:1113–20. 10.1038/leu.2010.54 20410923

[30] San Juan AF Fleck SJ Chamorro-Viña C Maté-Muñoz JL Moral S Pérez M. Effects of an intrahospital exercise program intervention for children with leukemia. Med Sci Sports Exerc. (2007) 39:13–21. 10.1249/01.mss.0000240326.54147.fc 17218878

[31] Thorsteinsson T Larsen HB Schmiegelow K Thing LF Krustrup P Pedersen MT. Cardiorespiratory fitness and physical function in children with cancer from diagnosis throughout treatment. BMJ Open Sport Exerc Med. (2017) 3:e000179. 10.1136/bmjsem-2016-000179 28761697 PMC5530132

[32] Shropshire J Carroll B Family variables and children's physical activity: influence of parental exercise and socio-economic status. Sport Educ Soc. (1997) 2:95–116. 10.1080/1357332970020106

[33] Gilliam MB Madan-Swain A Whelan K Tucker DC Demark-Wahnefried W Schwebel DC. Cognitive influences as mediators of family and peer support for pediatric cancer survivors' physical activity. Psychooncology. (2013) 22:1361–8. 10.1002/pon.3140 22826210 PMC3511656

